# Research priorities in the same-day and urgent care workforce: a priority-setting partnership in association with the James Lind Alliance

**DOI:** 10.1136/bmjopen-2026-126222

**Published:** 2026-11-09

**Authors:** Bethan Jones, Emily Phillpotts, Nahid Ahmad, Andy Gibson, Anna Katherine Mary Blackwell, Scott Watkins, Aloysius Niroshan Siriwardena, Uduak Archibong, Michael Clark, Sarah Voss, Nicola E Walsh

**Affiliations:** 1 School of Health and Social Wellbeing, University of the West of England, Bristol, UK; 2 University of Warwick, Coventry, UK; 3 Community and Health Research Unit, University of Lincoln, Lincoln, UK; 4 University of Bradford, Bradford, UK; 5 Care Policy and Evaluation Centre, The London School of Economics and Political Science, London, UK

**Keywords:** Health Workforce, ACCIDENT & EMERGENCY MEDICINE, Health Personnel, Health Services

## Abstract

**Abstract:**

**Objective:**

To identify and prioritise unanswered research questions that affect the UK urgent and emergency care (UEC) workforce from the perspectives of key informants including workforce groups and the public.

**Methods and analysis:**

This was a priority-setting partnership in association with the James Lind Alliance. The partnership used survey methods, WhatsApp data collection and online focus groups to identify indicative research questions from the UEC workforce, patients, carers and other interested parties. Literature checks confirmed that these questions had not previously been answered. The questions were prioritised via a survey and a final online workshop.

**Results:**

291 participants submitted 922 responses, which were analysed using qualitative content analysis to form 45 initial indicative questions. These were initially prioritised by 251 participants and 19 questions were taken to a final workshop in May 2026. This resulted in a top 10 list of unanswered research questions based on the perspective of workforce, patients and carers.

**Conclusion:**

Areas that were prioritised included questions on triage, communication, clinical decision making, risk and skill mix. These questions provide important directions for future research focusing on the UK UEC workforce and reflect their priorities.

**STRENGTHS AND LIMITATIONS OF THIS STUDY**

- The partnership engaged with multiple organisations aligned to under-represented groups in research.
- The use of multiple data collection methods supported broad engagement.
- This work used a robust and established method for identifying important unanswered questions and to then prioritise these.
- There were groups that remained under-represented, for example, responses appeared to be largely based in England.

## Introduction

### Background

 UK urgent and emergency care (UEC) services include ambulance response, urgent walk-in care (eg, urgent treatment centres), same-day general practice and community pharmacy and urgent community response services (eg, Frailty Assessment). The UEC workforce faces substantial operational and workforce challenges. The nature of these services means that professionals in this field are typically the first point of access for patients seeking healthcare. Increasing demand, seasonal fluctuations and constraints on patient flow across the wider health and care system exacerbate pressure.^1^ Additionally, these services have seen significant adaptation in recent years, including absorbing more responsibilities. This can all negatively impact mental and physical well-being; UEC staff have the highest sickness absence rates across the NHS.^2^ The psychological impact of pressure on staff well-being has also been highlighted in the 2025–2026 UEC Plan.^3^

The NHS Long Term Workforce Plan recognises that NHS staff are facing unprecedented levels of challenge.^4^ In UEC across the UK, these pressures are subject to the compounding effect of high staff turnover and staff shortages.^1 5–7^ It is important to understand the clinical operational and system-level challenges that exist across the broad and dynamic UEC sector to prioritise research where there are evidence gaps and support the development of a diverse thriving and sustainable workforce. Consulting members of the UEC workforce patients carers and the public is essential to identify the workforce challenges that arise in planning delivering and using services and to ensure that research addresses the questions that matter most. The James Lind Alliance (JLA) provides an established method for bringing these groups together in priority-setting partnership (PSPs) to identify and prioritise questions.^8^

Some existing PSPs in association with the JLA have covered aspects of the UK UEC workforce.^9 10^ However, these have primarily focused on clinical presentations, treatment protocols and patient experience. An adapted version of a JLA process, which produced themes that were adapted into indicative questions rather than directly collecting questions has been previously used to identify research questions with the workforce in primary care.^11^ This includes non-urgent care provision as well as same-day or urgent care aspects of general practice. The resulting priorities were arranged into three themes: working environment, service integration and the interface between patients and the workforce.

This is the first priority-setting exercise to identify research priorities across and within the UK UEC workforce. This is important because different sectors within UEC interact and impact each other. The UEC workforce in the UK is large, with staff working in a diverse range of roles and settings. There are a growing number of NHS professionals choosing portfolio careers or not working in a training scheme.^12^ Consequently, staff may be more likely to work across different services and identify cross-cutting challenges that warrant future research.

This PSP in association with the JLA was conducted as part of the portfolio of work delivered by the same-day and urgent care (SURGE) workforce research partnership.^13^ This national research partnership focuses on the UEC workforce across the NHS and social care in the UK. The findings of this PSP will both inform research projects undertaken by the SURGE workforce research partnership and will drive research more broadly in this field.

This paper has been written according to the reporting guidance for health research priority setting with stakeholders (REPRISE) checklist.^14^

## Methods

### Aim

The aim of the PSP was to identify and prioritise unanswered research questions that affect the UK UEC workforce from the perspectives of key informants including workforce groups and the public.

### Scope

The scope of UEC covered in this PSP focused on four key domains in the UK:

- Ambulance Services (including 999 and 111 call centres).
- Primary care, specifically general practice and community pharmacy.
- Emergency departments, specifically type one emergency departments and type three or four emergency departments (referred to as urgent treatment centres which can include GP out-of-hours services).
- Urgent community response services (eg, hospital at home services, virtual wards and mental health crisis teams).

The scope excluded questions about:

- UEC workforce outside the UK.
- NHS workforce not working in the UEC sector.
- Type two emergency departments, which focus on a single specialty, for example, dental, ophthalmology or maternity emergency care.

Participants in each data collection phase were clinical and non-clinical staff who worked in UK UEC services or patients and carers who used these services.

### Study design

The PSP was a mixed-methods study based on the JLA Priority Setting process. The JLA has a set of suggested processes with multiple phases that are described in detail in the guidebook.^8^ The analysis was led by a researcher acting as information specialist (BJ) in line with the guidebook. BJ has experience of qualitative research methods and surveys, and also acted as the PSP coordinator. The methods this PSP used are available in figure 1. As the PSP followed a multi-stage process, the methods and results have been combined for this paper to report the process chronologically.

**Figure 1:**
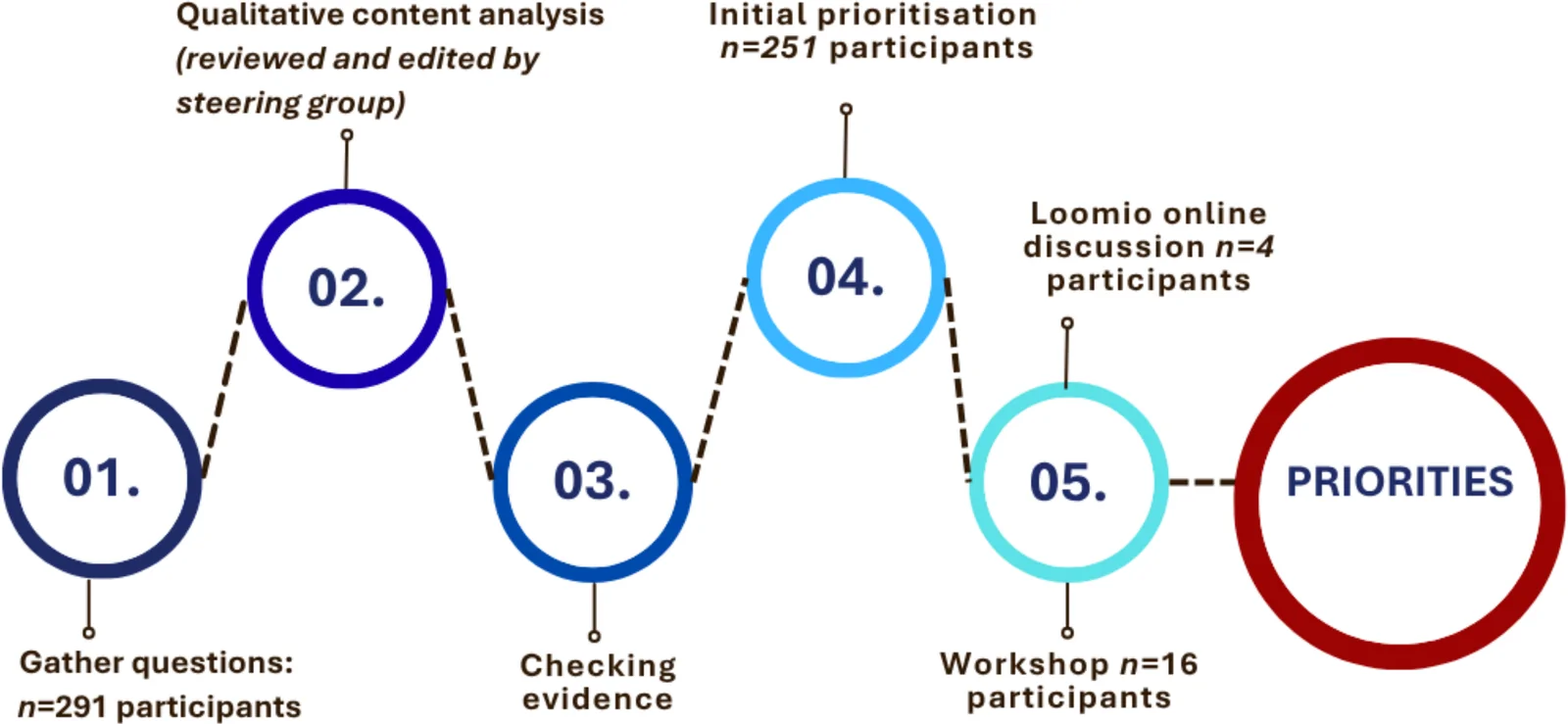
Overview schematic of JLA process.

The PSP methods were designed in consultation with two advisory panels (one for UEC professionals, one for patients and carers who have used these services) established to guide the broader workforce research partnership. These panels were different from the steering group (described below) and were recruited using established networks and contacts known to the partnership, through social media advertising and advertised through established PPI networks. Their role was to guide the design from the earliest stage of the project, including plans on recruiting a diverse steering group, methods to increase inclusivity for data collection and the scope of the project.

### Establishing a steering group: patient and public involvement and engagement

All JLA PSPs are guided and informed by a UK-wide collaborative steering group of staff, patients and carers and other relevant interested parties. This group is responsible for overseeing the work, informing decisions about the design and processes, reviewing the analysis and research questions and sharing recruitment materials and the final priorities.

The steering group for this PSP was assembled through proactive engagement with a range of organisations. This involved sending out an email flyer with an advertisement and a short video animation introducing the PSP and explaining the requirements. This was shared online on social media (BlueSky and LinkedIn), via authors’ existing networks and via national organisations and groups including staff networks, professional bodies and organisations representing staff in UEC, student groups in relevant fields and national diaspora associations. Members of the public with lived experience of UEC were contacted by circulating the opportunity via Research Engagement Networks (RENs), run by Integrated Care Boards across England. RENs focus on engaging with underserved communities. Using these networks helped us ensure diversity in our steering group. We supplemented this by contacting patient and public involvement and engagement networks in Wales, Scotland and Northern Ireland to ensure that we had a spread of people from across the UK. To monitor whether our recruitment process had been successful in accessing a diverse group of people interested in joining the steering group, respondents were asked to complete a brief expression of interest form, including optional demographic questions. This was to maximise variation in lived experience, geography and domains of the PSP scope across the group. For professional members, there was also variation in professions and in seniority by grade and years of experience.

The steering group was assembled in May 2025 and comprised 10 UEC professionals and four patient or carer representatives. In an adaptation of the typical JLA approach, the PSP steering group included more professional members than patients and carers due to the workforce focus of the PSP. The steering group met monthly online via Microsoft Teams, with meetings chaired by a JLA facilitator who advised the PSP (NA). The group approved the protocol, aims and scope of the project. The group also contributed to sharing the data collection materials and reviewed the data analysis. More information about the steering group is available in the protocol.^15^ Patient and carer group members were reimbursed for their time during meetings.

In conjunction with recruiting the steering group, we identified organisations to share the data collection and build awareness for the PSP. This thorough approach to engaging all interested parties was vital to ensure the prioritisation process was inclusive and robust, reflecting a wide-ranging set of experiences and perspectives.

### Data collection and analysis

#### Identifying questions

Recruitment targeted professionals working in UEC or people with experience of using these services as a patient or carer. Participants were recruited in the same way as the steering group (ie, social media, existing networks and by sharing details with organisations). The materials were shared with over 130 organisations, steering group members and other collaborators. All contacts were encouraged to share the links to take part to facilitate snowball sampling. Data collection for this phase took place between August and December 2025.

Potential participants were sent a flyer with a link to a landing page on the SURGE research website.^16^ Most PSPs typically use surveys in the first stage of data collection. However, we used an adapted approach to offer alternative methods to promote inclusivity and to ensure representation from people underserved by existing research. Participants were able to share questions and thoughts about important directions for future research by:

- Completing a Qualtrics survey (Provo, UT), which also collected optional demographic data about participants. This was monitored and used to identify under-represented groups to target for additional engagement as data were collected.
- Scanning a QR code to open an individual WhatsApp chat with the PSP to share ideas via voice note or in a written message.
- Completing a Microsoft Form to express an interest in attending an online focus group, between November and December 2025. These were separated into UEC professionals’ groups and patient and carer groups. Patients and carers were reimbursed for their attendance.

Participants were not offered incentives for taking part.

#### Participants

In total, 291 participants provided data, submitting a total of 922 questions or ideas. Of these, 241 completed the survey, 25 attended a focus group and 17 submitted a WhatsApp message or voice note. Eight participants did not submit an appropriate level of data for inclusion. This led to a total dataset from 283 participants.

Descriptive statistics on demographic data were monitored throughout data collection and used to summarise the sample. This information is available in table 1. Not all participants provided demographic data, and participants were able to select multiple responses in some items (eg, role, service worked, service used). As demographic questions were optional, it is not possible to know the full demographic characteristics of the sample. Representation varied between data collection phases, with some groups under-represented at different stages of the process.

**Table 1:** Demographic characteristics for data collection

	Identifying questions	Interim prioritisation	Final workshop
Total participants	283	251	20
Role			
Staff	156	138	16
Patient	78	74	2
Carer	34	32	2
Other/prefer not to say	27	32	0
Not stated	46	10	0
Staff sector			
Ambulance service	14	34	5
General practice or community pharmacy	58	34	6
Emergency department	72	59	6
Urgent community response	18	15	4
Not stated	4	6	0
Staff length of service			
<1 year	4	6	2
1–3 years	20	18	1
3–5 years	22	13	0
5–10 years	36	33	4
Over 10 years	69	60	7
Not stated	5	8	6
Patient/carer service used			
Ambulance service	48	57	Not published for confidentiality
GP or community pharmacy	53	59	
Emergency department	71	76	
Urgent community response	14	16	
Not stated	11	1	
Location			
England	195	150	16
Northern Ireland	3	15	1
Scotland	11	10	0
Wales	15	53	3
Not stated	59	23	0
Gender			
Man	81	74	6
Woman	131	148	14
Non-binary	2	4	0
Prefer not to say	11	3	0
Other	4	0	0
Not stated	54	22	0
Sexuality			
Straight/heterosexual	160	171	11
Bi/bisexual	11	20	2
Gay or lesbian	11	10	0
Pansexual	3	1	0
Asexual	3	6	0
Queer	2	2	0
Identify another way	22	19	4
Not stated	71	22	3
Experience as a carer			
Yes	87	82	8
No	130	140	10
Prefer not to say	14	7	2
Not stated	14	22	0
Lived experience of a disability	30	35	
Yes	187	188	1
No	2	0	14
Do not know	11	6	0
Prefer not to say	0	0	4
Not stated	53	21	0
Ethnicity			
White English/Welsh/Scottish/Northern Irish/British/Irish	172	182	13
Asian/Asian British	13	11	2
Black/Black British	2	3	0
Mixed/multiple ethnic groups	13	8	0
Other ethnic group - Arab	14	7	0
Prefer not to say	19	13	3
Not stated	50	22	2

#### Data cleaning and synthesis

Responses were synthesised into a single location for analysis. Free text data from the survey were extracted from the survey platform into a single Microsoft Excel spreadsheet. WhatsApp messages and voice notes were transcribed into the same spreadsheet, and the original chats were deleted. Field notes taken during each focus group, including each groups’ list of questions and topics to consider, were also integrated into the dataset. Subsequently, the dataset was reviewed for cleaning. Any potentially identifying information (eg, NHS trust or hospital names) were replaced with placeholder text. A small number of responses (n=8) were removed because they contained only placeholder text (eg, ‘.’ in all free text boxes) or demographic question responses only.

At this point, the information specialist (BJ) reviewed the data to identify any submissions that were potentially out of scope. As none of the responses had solely discussed issues that were out of scope with no wider relevance to the UK UEC workforce, the decision was taken to retain all the data and consider it to be in scope. This was discussed and agreed with the steering group, who had an opportunity to review the anonymised raw data.

#### Developing and refining questions

In line with the process described in the JLA guidebook,^8^ the next step was to categorise the raw survey data to form a list of indicative questions capturing the contents of the raw data, using Population, Concept, Context-style questions where possible. This was completed using conventional qualitative content analysis.^17^ This method was chosen because it offered the opportunity to identify patterns across the dataset by qualitatively coding the contents of each submission to identify features and ideas they contained. Each response was coded by attaching short labels that summarised the contents or features of the responses. These were compiled into 16 categories summarising the data. One person coded the data (BJ), and a second person (EP) reviewed the coding and added additional codes where necessary. Any remaining codes which did not align to a category were stored in a list of remaining codes. The categories covered broad concepts within the data, for example, codes related to skill mix or about training and professional development. Brief descriptions were then created for each category to describe the questions and ideas contained in the raw data related to each category and the boundaries of each category to avoid overlap wherever possible. This analysis necessitated repeated reading of the raw data to ensure that the analysis reflected the contents of the original submissions. Broad indicative questions were drafted to summarise the contents of the category.

The dataset was then read again to confirm that the list of questions represented the raw data and to add any questions that may not have been incorporated into categories but were present in the original data. This resulted in a list of 43 questions. The full coded dataset, category definitions and list of questions with extracts were all shared with the steering group for discussion and to approve the analysis.

The list of questions was discussed in detail with the steering group based on the JLA guidebook advice for finalising the list of questions.^8^ Some edits were made to the questions in this list based on a set of guiding principles the group agreed together. These edits included splitting larger questions into two and some rephrasing, resulting in a final list of 45 questions. These questions are available in Supplementary materials (‘online supplemental information 1: List of questions for interim prioritisation').

#### Checking evidence

Once questions were agreed, two PSP members (EP and BJ) conducted database and grey literature reviews for all questions to confirm that the questions identified were ‘true’ uncertainties before proceeding to prioritisation. This involved developing a search strategy (available in online supplemental materials 3: ‘search strategy’”) with input from a specialist librarian to review empirical papers and grey literature sources for evidence or guidance that adequately answered any of the existing questions.

The searches took place in^2^ and searched:

- MEDLINE.
- CINAHL.
- Grey literature sources including the websites of professional bodies for staff groups commonly working in UEC, think tanks such as the Kings Fund,^18^ the Health Foundation^19^ and the National Institute for Health Research evidence library.^20^

Across all these searches, a small number of questions had a relevant and recent systematic review that partially answered the question. For example, a partial answer related to a single staff group in a single context. No questions had been adequately answered, therefore no questions were removed from the list.

#### Interim prioritisation

A second survey was designed to prioritise the list of indicative questions. This was completed using Qualtrics (Provo, UT). The survey was sent to the organisations and networks that were contacted for the first round of data collection, along with other organisations and networks that had been identified in the interim period. Participants from the first round of data collection who signed up to receive updates about the PSP were also sent a link to the survey.

This second survey was a two-stage process. First, it asked participants to review the list of 45 questions and select all the questions they felt were important from the list of 45. The order of the list was randomised for each participant. Next, participants were presented with their shortlist of chosen questions. Participants were invited to choose their top 10 most important from their own shortlists. As with the first survey, optional demographics were available and these data were analysed weekly to target recruitment towards groups who were under-served by existing recruitment. As in the first phase of data collection, participants were not offered incentives for taking part.

A total of 251 participants completed this survey. This resulted in a list of prioritised questions to determine those that would be discussed in the final workshop. The 18 questions that were chosen most often across all participants combined were shortlisted for inclusion in the final workshop. These were discussed and approved by the steering group. Group rankings were not weighted differently, but not all participants disclosed their stakeholder group. Therefore, when separating out responses by group, the top question had been ranked first by staff and carers, fifth by patients, but also highly by those who did not disclose their relationship to UEC.

Initially, 18 questions were selected for discussion as this was considered to be a manageable number to discuss and rank based on the number of attendees and session lengths of the final workshop. Analysis also reviewed the most important questions by role (eg, staff, patient, carer) to ensure that the interests of all individual groups were well represented. This analysis identified a question that was sixth and seventh highest priority for patients and carers respectively but had not been included in the final workshop because its rank across the overall dataset was 22^nd^ (“What does continuity of care mean to the workforce in Urgent and Emergency Care?”). This was discussed with the steering group, who made the decision to include this additional question to ensure that such a high-priority question for both patients and carers was included.

The list of questions discussed at the workshop is included in table 2.

**Table 2:** List of questions included in final priority setting workshop

Question	Overall ranking	Staff ranking	Patient ranking	Carer ranking
What are the most appropriate approaches to triage in urgent and emergency care and how does this impact the workforce?	1	1	5	1
What are the staffing levels in urgent and emergency care and the consequences of this?	2	3	1	1
What services should be explored to enhance urgent and emergency care provision and how might these impact the workforce?	3	3	3	9
What is an appropriate skill mix for the urgent and emergency care workforce and in what context?	4	7	6	4
What does effectiveness and efficiency look like in urgent and emergency care workforce?	5	7	3	3
What factors affect the urgent and emergency care workforce when making clinical decisions?	6	3	6	4
What are the training gaps and needs across the urgent and emergency care workforce?	7	10	11	22
What training is needed to support the urgent and emergency care workforce to manage risk and uncertainty?	8	6	24	16
How does variation in clinical risk tolerance (individual and system-level) impact the workforce of urgent and emergency care?	9	2	35	9
How does availability of resources impact the workforce in urgent and emergency care?	10	14	6	9
How will Artificial Intelligence impact the urgent and emergency care workforce?	11	13	15	22
How does inter-professional communication affect the urgent and emergency care workforce?	12	12	24	7
How does rota/shift scheduling impact urgent and emergency care workforce well-being?	13	10	15	9
How can urgent and emergency care use staff skills most effectively?	14	9	15	28
What factors impact communication between patients and the urgent and emergency care workforce?	15	21	2	9
What are the drivers of staff retention and attrition in urgent and emergency care?	16	16	23	4
How does working in urgent and emergency care impact workforce well-being?	17	17	15	16
How does the physical environment impact the workforce in urgent and emergency care?	17	15	31	28
What does continuity of care mean to the workforce in urgent and emergency care?	22	21	6	7

### Final prioritisation workshop

The final half-day prioritisation online workshop took place in May 2026. This format was considered optimal to maximise opportunities for people working in UEC to attend. Participants for this event were recruited through completion of the interim prioritisation survey. Given the PSP focus on the workforce, most attendees were UEC professionals (n=12) and four were patients and/or carers. The PSP made efforts to ensure that all four of the sectors in the PSP scope were well represented in the final workshop. Similarly, we aimed for variation in demographic characteristics, profession and workplace setting. Patient or carer participants were offered a voucher at the NIHR standard participation rate for half a day. Professionals were not offered this incentive but were provided with a letter of participation for their professional development records.

The workshop was supplemented by online comments from four professionals who had expressed an interest but were unable to attend the workshop. This online discussion increased the number of participants who could contribute and offer an alternative, inclusive way to participate. This discussion occurred on Loomio,^21^ a consensus and decision-making platform. Here, participants were provided with the list of 19 questions and asked to identify the three highest and lowest priority questions for them and state reasons why. Data from discussion were incorporated into the first breakout discussion session at the final workshop.

In total, four people contributed on Loomio and 16 people attended the workshop. Demographic details for workshop attendees are available in table 1. The workshop involved three discussion sessions to rank the questions and review aggregated rankings across several breakout sessions facilitated by experienced JLA facilitators. This is based on an adaptation of Nominal Group Technique,^8^ which aims to balance views across participants in a group and avoid hierarchy in decision making. The first session focused on asking participants to share their three highest and three lowest ranked questions based on their own individual prioritisation (completed in advance of the workshop) and Loomio rankings were incorporated at this point. This was followed by two more sessions of discussion and ranking.

#### Top 10

The final ranking of the questions is listed in order in table 3. The ranking of all 19 questions is available in Supplementary materials (‘online supplemental information 2: Ranking of 19 questions in final workshop’).

**Table 3:** Final top 10 priorities in order

1	What are the most appropriate approaches to triage in urgent and emergency care and how does this impact the workforce?
2	What factors impact communication between patients and the urgent and emergency care workforce?
3	What factors affect the urgent and emergency care workforce when making clinical decisions?
4	How might services be enhanced in urgent and emergency care and how would this impact the workforce?
5	What is an appropriate skill mix for the urgent and emergency care workforce and in what context?
6	How does variation in clinical risk tolerance (individual and system-level) impact the workforce of urgent and emergency care?
7	What training is needed to support the urgent and emergency care workforce to manage risk and uncertainty?
8	How does inter-professional communication affect the urgent and emergency care workforce?
9	What are the training gaps and needs across the urgent and emergency care workforce?
10	How will Artificial Intelligence impact the urgent and emergency care workforce?

## Discussion

The purpose of this PSP was to identify and prioritise unanswered research questions about the UK UEC workforce from the perspectives of key stakeholder groups. Triage was the highest priority question in the final workshop and a commonly mentioned issue in the question-gathering stage. NHS UEC services use a range of models and approaches, including a growing use of algorithms which has been found to be more risk averse compared with health professional triage.^22 23^ In primary care, evidence indicates that triage is commonly conducted by nurses but there is substantial variation in approaches.^24^ Triage is a system-wide challenge that impacts task allocation, workload and training; variation in approaches will have implications for the workforce within these services.^24 25^ Therefore, risk averse triage can lead to the additional strains on services and bottlenecks in patient flow described in the introduction. Triage can also be shaped by demands or pressures on the system.

Two of the indicative questions covered aspects of risk tolerance and managing uncertainty (with 11 responses that contributed to the development of these questions). Risk is inherent within UEC where patients frequently present with undifferentiated illness and clinicians and services must continually balance competing risks when making decisions; therefore, both individuals and different services must be prepared to manage this uncertainty. As evidence indicates that tolerance of uncertainty is associated with health professional well-being,^26^ this will have a wider impact on the workforce. Over- and under-treating due to risk tolerance may also impact pressures on the system.

There are a growing number of non-medical roles in the UEC workforce. Skill mix was addressed in the prioritised questions: “What is an appropriate skill mix for the Urgent and Emergency Care workforce and in what context?”. There were forty responses to the initial survey that contributed to this question. However, additional responses relating to other issues (eg, scope of practice) also captured issues relevant to skill mix. For example, “What is considered to be a first contact practitioner’s scope of practice?” was a question submitted in the initial data collection and an example of questions relevant to skill mix. In total, skill mix was relevant to 83 initial responses. The Long-term Workforce Plan^4^ proposes developing skill mix through routes like apprenticeships and advanced practice opportunities. Newer roles being integrated into the workforce and this has implications for workforce planning, supervision and task allocation.^27^ However, in UEC, with staff shortages and high turnover,^5^ it is particularly important to understand the impact of different roles on team working and service delivery. Understanding skill mix and organisational structure is important to make best use of staff skills that are important for the delivery of all UEC services.

The process adopted during this PSP was an adapted approach. Instead of including questions proposed by participants verbatim, the data collection process enabled us to identify a broad range of issues and ideas which were iteratively synthesised into research questions. This adapted approach was adopted because this PSP differs due to its focus on the workforce across different sectors of UEC rather than a specific patient condition or discrete intervention. The aim was to identify challenges and issues that were common across different UEC services.

Some findings were similar to those identified by PSPs that focused on more discrete parts of the healthcare system (eg, general practice, paediatric emergency medicine) including questions about interacting with patients, triage and communication between services.^10 11^ However, other findings reflect the challenges related to a workforce that operates in a complex UEC system where availability and efficiency on one service impacts the demand on others. This results in service and system adaptations to manage pressure and meet increasing demand. One example of this is an initiative to reduce avoidable admissions with ‘call before convey’ schemes.^28^ These use inter-professional communication to provide a service enhancement, requiring shared risk tolerance between individuals and services. Similarly, AI tools are increasingly being implemented to stream or prioritise calls^3^ which is intended to improve patient flow in the face of increased demand on staff.

There were three questions that the research team expected to be important that were not prioritised at the interim stage. These were questions on recruitment (What enables future workforce recruitment in Urgent and Emergency Care?), retention (How does training impact retention in the Urgent and Emergency Care workforce?) and scope of practice (“What contributes to variation in scope of practice in the Urgent and Emergency Care workforce?”). There was also a noticeable lack of questions about role satisfaction and initiatives to improve staff engagement. This may be because the majority of responses were from frontline staff and the issues they were most concerned about were those more directly impacting day-to-day work (eg, staffing levels, triage, shift-scheduling and the use of Artificial Intelligence). These factors are likely to be exacerbated by the pressures and challenges described in the introduction, meaning that questions about effectiveness, resourcing and service provision feel more urgent and immediate for workforce and patients.

This work is strengthened by embedding significant patient and public involvement into the work. The PSP was guided by the steering group discussed above and the broader patient and professional groups described in the method section. Another strength of this PSP was the commitment to equality, diversity and inclusion adopted throughout the process. Reliance on survey methods may result in response bias. In order to address this, we adopted inclusive methods to data collection (WhatsApp and focus groups), targeted a wide range of networks and organisations for recruitment and applied practical adaptations during the steering group meetings including recording summaries of data analysis for group members to review at a pace that best suited them.

Despite efforts to recruit a diverse sample with good representation across a range of characteristics and professions, some groups of people remain under-represented at all stages of the PSP according to the demographic data collected. For example, ambulance services staff were under-represented in the first round of data collection and small numbers of professionals working in urgent community response services took part throughout. While the cross-sector approach to data collection allowed the opportunity to identify shared priorities across different services and sectors in UEC, this resulted in questions that, at times, were very broad in scope and phrasing to be relevant across the sectors.

Future research is now needed to address these 10 priorities, as well as the other research questions identified throughout this PSP as there are no current plans to refresh or update this list at this time. Some of this work will be undertaken by the SURGE workforce research partnership. The priority list has been disseminated to funders and the groups and networks that shared the recruitment materials, as well as to academic, professional and public audiences and we have a knowledge mobilisation plan to track impact. This will involve the networks and connections established throughout the PSP to ensure wide reach.

## Conclusion

This PSP used meaningful and extensive engagement strategies to identify relevant UK UEC workforce research priorities. The JLA process, along with adaptations to best suit the population, provided a structured approach to identifying a shortlist of high-priority research questions. Future research should engage with these priorities to develop the evidence needed to ensure a sustainable UK UEC workforce for the future.

### Protocol

The original protocol for this study is available on the James Lind Alliance website at https://www.jla.nihr.ac.uk/documents/same-day-and-urgent-care-surge-workforcepsp-protocol

## Supplementary material

10.1136/bmjopen-2026-126222 online supplemental file 1

10.1136/bmjopen-2026-126222 online supplemental file 2

10.1136/bmjopen-2026-126222 online supplemental file 3

## Data Availability

Data are available upon reasonable request.

## References

[1] Welsh Government (2022). Right care, right place, first time six goals for urgent and emergency care six goals for urgent and emergency care.

[2] NHS England (2026). NHS sickness absence rates, February 2026 [Internet]. https://digital.nhs.uk/data-and-information/publications/statistical/nhs-sickness-absence-rates/february-2026.

[3] NHS England (2025). Urgent and Emergency Care Plan 2025/26 [Internet]. https://www.england.nhs.uk/long-read/urgent-and-emergency-care-plan-2025-26.

[4] NHS England (2023). NHS long term workforce plan [internet]. https://www.england.nhs.uk/wp-content/uploads/2023/06/nhs-long-term-workforce-plan-v1.21.pdf.

[5] Winchester N (2023). Emergency healthcare in crisis: Public Services Committee report. House of Lords Library [Internet].

[6] Health and Social Care Northern Ireland (2016). HEALTH and social care workforce strategy 2026 [internet]. https://www.health-ni.gov.uk/sites/default/files/publications/health/hsc-workforce-strategy-2016.pdf.

[7] Royal College of Emergency Medicine (2021). Scotland’s emergency medicine workforce census [internet]. https://rcem.ac.uk/wp-content/uploads/2021/10/Scotlands_Emergency_Medicine_Workforce_Census.pdf.

[8] (2021). The James Lind Alliance Guidebook [Internet].

[9] Cottey L, Shanahan TAG, Gronlund T (2023). Refreshing the emergency medicine research priorities. Emerg Med J.

[10] Sloane C, Waterfield T, Evans J (2026). Refreshing the paediatric emergency medicine research priorities across the UK and Ireland. Emerg Med J.

[11] Abrams R, Blake S (2024). UK general practice service delivery research priorities: an adapted James Lind Alliance approach. Br J Gen Pract.

[12] Burnham V, Varughese GI (2024). Locally employed doctors and international medical graduates in the NHS: current and evolving trends. Br J Hosp Med.

[13] National Institute for Health Research (2025). Same day and urgent care workforce research partnership (SURGE) [Internet]. https://fundingawards.nihr.ac.uk/award/NIHR159864.

[14] Tong A, Synnot A, Crowe S (2019). Reporting guideline for priority setting of health research (REPRISE). BMC Med Res Methodol.

[15] (2025). Same Day and Urgent Care (SURGE) Workforce PSP Protocol [Internet]. https://www.jla.nihr.ac.uk/documents/same-day-and-urgent-care-surge-workforce-psp-protocol.

[16] (2026). SURGE workforce partnership [internet]. https://surgeworkforce.uk.

[17] Hsieh H-F, Shannon SE (2005). Three approaches to qualitative content analysis. Qual Health Res.

[18] The King's Fund [Internet]. https://www.kingsfund.org.uk.

[19] The Health Foundation [Internet]. https://www.health.org.uk.

[20] NIHR Evidence [Internet]. https://evidence.nihr.ac.uk.

[21] Loomio [Internet]. https://loomio.eu.

[22] Podlasek A, Sanders T, Payne RE (2026). What do we mean by urgent care? Definitional confusion and its consequences. *Br J Gen Pract*.

[23] Chambers D, Cantrell AJ, Johnson M (2019). Digital and online symptom checkers and health assessment/triage services for urgent health problems: systematic review. BMJ Open.

[24] Park HN, Lee K, Wallace C (2026). The use of triage in primary care in the UK: an integrative review and narrative synthesis. J Adv Nurs.

[25] Gorick H, McGee M, Smith T (2025). Understanding the demographics, training experiences and decision-making practices of UK triage nurses. Emerg Nurse.

[26] Budworth L, Wilson B, Sutton-Klein J (2024). Is emergency doctors’ tolerance of clinical uncertainty on a novel measure associated with doctor well-being, healthcare resource use and patient outcomes?. Emerg Med J.

[27] McDermott I, Spooner S, Goff M (2022). Scale, scope and impact of skill mix change in primary care in England: a mixed-methods study. *Health Soc Care Deliv Res*.

[28] Noble S, Flattery P, Stonehouse W (2046). Call before convey: delivering urgent care for patients in the right place with the right clinician, first time. Emerg Med J [Internet].

