# Supplementary material for: Research priorities in the same-day and urgent care workforce: a priority-setting partnership in association with the James Lind Alliance
Source: BMJ Open. 2026 Nov 9;16(11):e126222. doi: 10.1136/bmjopen-2026-126222 (PMC13756369; doi:10.1136/bmjopen-2026-126222)
Supplement: Supplementary file 1 [file bmjopen-2026-126222.reviewer_comments.pdf]

PEER REVIEW HISTORY

BMJ Open publishes all reviews undertaken for accepted manuscripts. Reviewers are asked to complete a checklist review form and are provided with free text boxes to elaborate on their assessment. These free text comments are reproduced below.

ARTICLE DETAILS

Title (Provisional)

Research priorities in the same day and urgent care workforce: A priority setting partnership in association with the James Lind Alliance

Authors

Jones, Bethan; Phillpotts, Emily; Ahmad, Nahid; Gibson, Andy; Blackwell, Anna Katherine Mary; Watkins, Scott; Siriwardena, Aloysius Niroshan; Archibong, Uduak; Clark, Michael; Voss, Sarah; Walsh, Nicola E

VERSION 1 - REVIEW

**Reviewer**	**1**
**Name**	**Kumlien, Christine**
**Affiliation**	**Malmö Universitet, Care science**
**Date**	**21-Aug-2026**
**COI**	**None**

This is an important paper describing the JLA process to identify the top 10 research uncertainties of importance for the UK urgent and emergency care workforce. The result provides important information for researchers and can guide future research within the topic. The manuscript is in general well written, however, there some aspects that need to be clarified before publication. Thank you for letting me review this paper and I enjoyed the reading.

Abstract

The aim in the abstract, state that it is only about the UK urgent and emergency care workforce which but in the manuscript the aim is to identify and prioritise unanswered research questions about the UEC workforce in general terms. Good if both aims corresponding.

Introduction

The introduction is explaining about the challenges that UK urgent and emergency care workforce are facing right now. It gives a clear direction that this paper is about problems,

constraints and demands for the professionals working in the urgent and emergency care. However, the aim is more generally expressed.

Aim

The aim to identify and prioritise unanswered research questions about the UEC workforce is very broad and unspecific. The identified research questions concern triage, communication (interprofessional and with patients), clinical decision making, services, skills and training, risk tolerance and AI. A more proprietary aim could be to identify and prioritise unanswered research questions about areas or factors that influence/affects the UEC workforce (to improve service delivery or improve work conditions).

Methods

The JLA process is in general clear described and easy to follow. However, there are some unclarities that need to be explained more in detail.

On page 7 I can read that 291 participants provided data but then later the authors state that 8 participants did not submit an appropriate level of data and were excluded. Should the number of participants be 283 instead?

Table 1 is not clear even if the authors explain that the demographic questions were not optional and it is therefore not possible to know the demographic characteristics. A suggestion is to state the original number of participants in the different phases of the data collection. It would also be good to see how many of the participants that chose not to answer these questions.

Discussion

The discussion is handling the different the top 10 research questions just on a surface level and does not connect them to any of the challenges that are mentioned in the introduction.

On page 15 line -2, it is written that several questions covered aspects of risk tolerance and managing uncertainty. How many are several? I have only identified two. Please give some examples.

Same thing regarding skill mix and the growing number of non-medical roles where it is written that this was discussed in many questions. Please give examples. Only one question concern skill mix.

I miss a discussion about the fact that no question concerned things like initiatives that can improve staff engagement, resilience, retention, satisfaction, individuality and responsibility. According to the introduction this must be most relevant questions for the UEC workforce.

**Reviewer**	**2**
**Name**	**Norris, Sarah**
**Affiliation**	**Newcastle Upon Tyne Hospitals NHS Foundation Trust**

**Date**	**16-Sep-2026**
**COI**	**None**

Thank you for your submission. Overall, this was clearly written with good structure. I would suggest the following minor changes, for clarity:

Please ensure consistent referencing (e.g. p.4 lines 30-31)

It would be useful to add a sentence to explain how the JLA adapted process is different from the JLA standard process (p.4, line 48)

"The PSP methods were also designed in consultation with two panels (one for UEC professionals, one for patients and carers) established to guide the broader workforce research partnership." (p. 6, lines 16-19) would be better placed elsewhere, with additional detail (e.g. formation/scope of the panels, or citations). This would improve clarity by differentiating these panels from the steering group.

Add initials for coders (p.11, line 10).

Please add your search strategies used to verify if the interim questions had been answered, as supplemental file.

Please clarify if/how rankings were weighted (p.11, lines 43-52), as this in unclear from this paragraph and Table 2. E.g. Overall ranking of 1 given to "What are the most appropriate approaches to triage in Urgent and Emergency Care and how does this impact the workforce?" (1+5+1=7) when "What are the staffing levels in Urgent and Emergency Care and the consequences of this?" appears to rank higher with a lower overall score (3+1+1=5).

Please update to show how your manuscript meet the REPRISE guidelines in the REPRISE table (p. 25).

VERSION 1 - AUTHOR RESPONSE

Revision requested	Author response
The aim in the abstract, state that it is only about the UK urgent and emergency care workforce which but in the manuscript the aim is to identify and prioritise unanswered research questions about the UEC workforce in general terms. Good if both aims corresponding.	We have rephrased the objective in the abstract and the aim in the main manuscript so that they align. We have clarified that we are referring to the UK UEC workforce throughout the manuscript.
The aim to identify and prioritise unanswered research questions about the UEC workforce is very broad and unspecific. The identified research questions concern triage, communication (interprofessional and with patients), clinical decision making, services, skills and training, risk	The study aim has been rephrased to clarify that this work is focussed on issues affecting the UK UEC workforce.

tolerance and AI. A more proprietary aim could be to identify and prioritise unanswered research questions about areas or factors that influence/affects the UEC workforce (to improve service delivery or improve work conditions).	
On page 7 I can read that 291 participants provided data but then later the authors state that 8 participants did not submit an appropriate level of data and were excluded. Should the number of participants be 283 instead?	A sentence has been added to confirm that data from a total of 283 participants were included.
Table 1 is not clear even if the authors explain that the demographic questions were not optional and it is therefore not possible to know the demographic characteristics. A suggestion is to state the original number of participants in the different phases of the data collection.	We have added a row to table 1 to indicate the total of participants in each phase of data collection.
It would also be good to see how many of the participants that chose not to answer these questions.	We have clarified how many participants did not respond to each of the demographic questions at each stage.
The discussion is handling the different the top 10 research questions just on a surface level and does not connect them to any of the challenges that are mentioned in the introduction.	We have gone into more detail in the discussion, including adding additional text about questions that were and were not prioritised, and adding more on workforce pressure in response to service and system adaptations that have been introduced to try and meet demand and their relation to the top 10 questions.
On page 15 line -2, it is written that several questions covered aspects of risk tolerance and managing uncertainty. How many are several? I have only identified two. Please give some examples.	We have clarified here that we refer to two of the overall indicative questions. However, we have also mentioned that 11 responses contributed to these two questions.
Same thing regarding skill mix and the growing number of non-medical roles where it is written that this was discussed in many questions. Please give examples. Only one question concern skill mix.	We have specified one question and explained that 83 initial submissions were relevant to skill mix.
I miss a discussion about the fact that no question concerned things like initiatives that can improve staff engagement, resilience, retention, satisfaction, individuality and responsibility.	We have noted this in the discussion, suggested some potential contributors to this and related it to the pressures and

According to the introduction this must be most relevant questions for the UEC workforce.	challenges described in the introduction.
Please ensure consistent referencing (e.g. p.4 lines 30-31)	Thank you for flagging this error in using our citation software, we have updated the referencing for consistency.
It would be useful to add a sentence to explain how the JLA adapted process is different from the JLA standard process (p.4, line 48)	We have explained how that study's approach differed from the standard JLA process.
"The PSP methods were also designed in consultation with two panels (one for UEC professionals, one for patients and carers) established to guide the broader workforce research partnership." (p. 6, lines 16-19) would be better placed elsewhere, with additional detail (e.g. formation/scope of the panels, or citations). This would improve clarity by differentiating these panels from the steering group.	We have relocated this sentence to the study design section. We have also provided additional detail on the formation and scope of these additional groups to differentiate them from the steering group.
Add initials for coders (p.11, line 10).	Initials for the coders have been added.
Please add your search strategies used to verify if the interim questions had been answered, as supplemental file.	Supplementary information file 3 contains the search strategy used to confirm that the interim questions had not already been answered.
Please clarify if/how rankings were weighted (p.11, lines 43-52), as this in unclear from this paragraph and Table 2. E.g. Overall ranking of 1 given to "What are the most appropriate approaches to triage in Urgent and Emergency Care and how does this impact the workforce?" (1+5+1=7) when "What are the staffing levels in Urgent and Emergency Care and the consequences of this?" appears to rank higher with a lower overall score (3+1+1=5).	We have clarified that the rankings were not weighted by group and that we initially chose 18 questions based on overall response. However, an additional question (What does continuity of care mean to the workforce in Urgent and Emergency Care?) was included because a question that was important to patients and carers was not initially in the top 18 questions. We have also explained how the overall ranking was also determined by participants who did not disclose whether they were staff, patient, or carer. Therefore, they were not represented in the subgroup analysis but did contribute to the overall prioritisation.

Please update to show how your manuscript meet the REPRISE guidelines in the REPRISE table (p. 25).	We have edited this table to note where each section is presented in the manuscript. This has resulted in several additions to be sure we adhere to the guidance<br>1. Referring to BJ as information specialist and describing experience for this under Study Design<br>2. Noting that other than patient/carer participants in the final workshop, participants did not receive incentives or compensation for taking part<br>3. Referring to the knowledge mobilisation plan for tracking and evaluating impact<br><br>Thank you for the suggestion to review this and improve the report.
