# Supplementary material for: Research priorities in the same-day and urgent care workforce: a priority-setting partnership in association with the James Lind Alliance
Source: BMJ Open. 2026 Nov 9;16(11):e126222. doi: 10.1136/bmjopen-2026-126222 (PMC13756369; doi:10.1136/bmjopen-2026-126222)
Supplement: Supplementary file 2 [file bmjopen-16-11-s001.docx]

### Supplementary information 1: List of questions for interim prioritisation

- What are the most appropriate approaches to triage in Urgent and Emergency Care and how does this impact the workforce?
- What are the staffing levels in Urgent and Emergency Care and the consequences of this?
- What services should be explored to enhance UEC provision, and how might these impact the workforce?
- What is an appropriate skill mix for the Urgent and Emergency Care workforce and in what context?
- What does effectiveness and efficiency look like in Urgent and Emergency Care workforce?
- What factors affect the Urgent and Emergency Care workforce when making clinical decisions?
- What are the training gaps and needs across the Urgent and Emergency Care workforce?
- What training is needed to support the Urgent and Emergency Care workforce to manage risk and uncertainty?
- How does variation in clinical risk tolerance (individual and system-level) impact the workforce of Urgent and Emergency Care?
- How does availability of resources impact the workforce in Urgent and Emergency Care?
- How will AI impact the Urgent and Emergency Care workforce?
- How does inter-professional communication affect the Urgent and Emergency Care workforce?
- How does rota/shift scheduling impact Urgent and Emergency Care workforce wellbeing?
- How can Urgent and Emergency Care use staff skills most effectively?
- What factors impact communication between patients and the Urgent and Emergency Care workforce?
- What are the drivers of staff retention and attrition in Urgent and Emergency Care?
- How does working in Urgent and Emergency Care impact workforce wellbeing?
- How does the physical environment impact the workforce in Urgent and Emergency Care?
- What enables Urgent and Emergency Care services and staff to anticipate and respond to demand?
- How does digitalisation and the use of technology impact clinical decision making in Urgent and Emergency Care?
- To what extent are efforts duplicated in Urgent and Emergency Care, and what are the consequences of this for the workforce?
- What does continuity of care mean to the workforce in Urgent and Emergency Care?
- What interventions are effective in supporting Urgent and Emergency care staff and services to manage demands on services?
- What is the role and impact of remote management in Urgent and Emergency Care workforce?
- What support is available and effective for Urgent and Emergency Care workforce wellbeing?
- What encourages the workforce to join and remain in Urgent and Emergency Care?
- What approach to leadership best supports the Urgent and Emergency Care workforce?
- What are the drivers and consequences of demand across different parts of the urgent and emergency care system for staff?
- How does the availability of IT infrastructure support the workforce
- What contributes to variation in scope of practice in the Urgent and Emergency Care workforce?
- How older members of the workforce be supported to stay working in Urgent and Emergency Care?
- What training/support is needed for the Urgent and Emergency Care workforce to deliver culturally competent and/or inclusive care?
- What enables future workforce recruitment in Urgent and Emergency Care?
- What are the consequences of different approaches to skill mix across the Urgent and Emergency Care workforce, including gaps in the workforce?
- What are the barriers and facilitators to progressional development in Urgent and Emergency Care?
- What are the Urgent and Emergency Care workforces experiences of abuse in their roles?
- What are the workforce consequences of different approaches to making clinical decisions in Urgent and Emergency Care?
- In what ways might workforce in one part of the Urgent and Emergency Care system impact workforce in other parts of the system?
- How can Urgent and Emergency Care professionals with caring responsibilities be supported in the workforce?
- What factors impact recruitment and retention of international staff in Urgent and Emergency Care?
- What are the consequences of increased digitalisation for Urgent and Emergency Care staff?
- How does training impact retention in the Urgent and Emergency Care workforce?
- What is the workforce impact of different models of team working across Urgent and Emergency Care?
- What is the workforce impact of organisational culture in Urgent and Emergency Care?
- How does cross-sector working affect the Urgent and Emergency Care workforce?
