# Supplementary material for: Research priorities in the same-day and urgent care workforce: a priority-setting partnership in association with the James Lind Alliance
Source: BMJ Open. 2026 Nov 9;16(11):e126222. doi: 10.1136/bmjopen-2026-126222 (PMC13756369; doi:10.1136/bmjopen-2026-126222)
Supplement: Supplementary file 3 [file bmjopen-16-11-s002.docx]

### Supplementary information 2: Ranking of 19 questions in final workshop

| 1 | What are the most appropriate approaches to triage in Urgent and Emergency Care and how does this impact the workforce? |
| --- | --- |
| 2 | What factors impact communication between patients and the Urgent and Emergency Care workforce? |
| 3 | What factors affect the Urgent and Emergency Care workforce when making clinical decisions? |
| 4 | How might services be enhanced in Urgent and Emergency Care, and how would this impact the workforce? |
| 5 | What is an appropriate skill mix for the Urgent and Emergency Care workforce and in what context? |
| 6 | How does variation in clinical risk tolerance (individual and system-level) impact the workforce of Urgent and Emergency Care? |
| 7 | What training is needed to support the Urgent and Emergency Care workforce to manage risk and uncertainty? |
| 8 | How does inter-professional communication affect the Urgent and Emergency Care workforce? |
| 9 | What are the training gaps and needs across the Urgent and Emergency Care workforce? |
| 10 | How will AI impact the Urgent and Emergency Care workforce? |
| 11 | What does effectiveness and efficiency look like in Urgent and Emergency Care workforce? |
| 12 | How does working in Urgent and Emergency Care impact workforce wellbeing? |
| 13 | What are the drivers of staff retention and attrition in Urgent and Emergency Care? |
| 14 | How can Urgent and Emergency Care use staff skills most effectively? |
| 15 | How does availability of resources impact the workforce in Urgent and Emergency Care? |
| 16 | What are the staffing levels in Urgent and Emergency Care and the consequences of this? |
| 17 | What does continuity of care mean to the workforce in Urgent and Emergency Care? |
| 18 | How does rota/shift scheduling impact Urgent and Emergency Care workforce wellbeing? |
| 19 | How does the physical environment impact the workforce in Urgent and Emergency Care? |
