# Supplementary material for: Research priorities in the same-day and urgent care workforce: a priority-setting partnership in association with the James Lind Alliance
Source: BMJ Open. 2026 Nov 9;16(11):e126222. doi: 10.1136/bmjopen-2026-126222 (PMC13756369; doi:10.1136/bmjopen-2026-126222)
Supplement: Supplementary file 4 [file bmjopen-16-11-s003.docx]

Supplementary information: Search strategy

urgent care OR emergency care OR same day care OR general practice OR primary care OR pre hospital care OR pre-hospital care OR out of hospital care OR out-of-hospital care OR ambulatory OR crisis OR critical

AND NHS

AND workforce OR staff OR paramedic OR nurs* OR doctor* OR physician* OR general practi* OR physio* OR pharmacist OR advanced practi* OR AHP OR allied health prof*

AND review OR meta analys* OR metaanalys* OR meta synthes* OR meta-synthes* OR meta-analys*
